# Impact of AFM-induced nano-pits in a-Si:H films on silicon crystal growth

**DOI:** 10.1186/1556-276X-6-145

**Published:** 2011-02-15

**Authors:** Elisseos Verveniotis, Bohuslav Rezek, Emil Šípek, Jiří Stuchlík, Martin Ledinský, Jan Kočka

**Affiliations:** 1 Institute of Physics ASCR, Cukrovarnicka 10, 16253, Prague 6, Czech Republic

## Abstract

Conductive tips in atomic force microscopy (AFM) can be used to localize field-enhanced metal-induced solid-phase crystallization (FE-MISPC) of amorphous silicon (a-Si:H) at room temperature down to nanoscale dimensions. In this article, the authors show that such local modifications can be used to selectively induce further localized growth of silicon nanocrystals. First, a-Si:H films by plasma-enhanced chemical vapor deposition on nickel/glass substrates are prepared. After the FE-MISPC process, yielding both conductive and non-conductive nano-pits in the films, the second silicon layer at the boundary condition of amorphous and microcrystalline growth is deposited. Comparing AFM morphology and current-sensing AFM data on the first and second layers, it is observed that the second deposition changes the morphology and increases the local conductivity of FE-MISPC-induced pits by up to an order of magnitude irrespective of their prior conductivity. This is attributed to the silicon nanocrystals (<100 nm) that tend to nucleate and grow inside the pits. This is also supported by micro-Raman spectroscopy.

## Introduction

Crystallization of amorphous silicon (a-Si:H) films is traditionally employed as an alternative method for producing large-area electronics such as displays and solar cells. It is typically induced by laser [1] or high-temperature furnace annealing [2]. The presence of silicide-forming metals such as nickel [3] or the application of an electric field [4,5] was found to reduce the crystallization temperature.

Nowadays, the production of silicon nanocrystals has become increasingly important as they are attractive for nanoelectronic, optoelectronic, as well as biological applications [6]. Usually, they are produced in the form of the so-called micro-crystalline silicon thin films using chemical vapor deposition (CVD) [7,8] or by electrochemical etching of bulk monocrystalline silicon, yielding the so-called porous silicon [9]. Yet, producing the nanocrystals in well-defined locations or creating arranged microscopic patterns still remains a challenging task.

Recently, our previous studies have shown that field-enhanced [4,5] metal-induced [3] solid-phase crystallization (FE-MISPC) at room temperature can be used to achieve spatially localized current-induced crystallization of a-Si:H films using a sharp tip such as those employed in atomic force microscopy (AFM) [10]. This process resulted in the formation of microscopic crystalline rings and dots as well as resistive nano-pits at controlled positions in the a-Si:H thin films. The smallest sizes of the crystallized objects ranged from a few hundred nanometers to several micrometers due to electrical discharge from the inherently present parallel capacitance, caused by a drastic increase of local material conductivity (and hence a decrease of potential difference on the parallel capacitance) after the dielectric breakdown of the films. The process was then further miniaturized below 100 nm by limiting the passing current (which was fluctuating below a given set-point) and thus also the electrical discharge between the conductive AFM tip and bottom nickel electrode [11]. On the other hand, perfectly stabilized electrical current during FE-MISPC process produced mainly non-conductive pits [12].

In this study, how the FE-MISPC-induced features (conductive and non-conductive pits) affect further nucleation and growth of the secondary silicon thin film is investigated. For this purpose, the second silicon layer at the boundary condition of amorphous/micro-crystalline growth after local FE-MISPC modifications of the first fully amorphous layer is deposited. The effects of the second deposition on the crystallinity, conductivity, structure, and spatial localization of the features based on their initial morphology and conductivity are discussed.

## Method

The a-Si:H films are deposited by plasma-enhanced CVD in a thickness of 170 nm (±30 nm, measured by a stylus profilometer) on a Corning 7059 glass substrate coated with 40-nm-thin nickel film and 10 nm titanium interlayer for improved adhesion to glass. Substrate temperature of 50°C and 0.02% dilution of SiH_4 _in helium result in a hydrogen content of 20-45 at.% in the films [13].

The FE-MISPC is accomplished by applying the electric field locally using a sharp conductive tip in AFM. Employed tips were either Pt/Cr-coated doped silicon (ContE, Budgetsensors) or conductive diamond-coated silicon (DCP11, NT-MDT). The typical tip radius is 10-70 nm depending on the type used. The tips are put in contact with the a-Si:H film with the force of 10-500 nN. The current source is connected to the bottom nickel electrode. The nickel electrode is negatively biased to facilitate the FE-MISPC process [4]. Oxidation of the silicon surface is thus of no concern as the AFM tip polarity cannot give rise to local anodic oxidation [14]. Details of the setup can be found in Refs. [11,12]. The FE-MISPC process is realized by a sample current of -0.5 nA, which is part of the constant current (-100 nA) applied by an external source unit (Keithley K237). Outcome of the exposition is determined by its temporal profile [12].

Microscopic morphology and local conductivity of the films before and after the FE-MISPC process are characterized by current-sensing AFM (CS-AFM) [15] using sample bias voltage of -25 V. Increased local current detected by CS-AFM is a good indication of crystallinity as corroborated previously by micro-Raman spectroscopy [11]. Such high sensing bias is used because of the amorphous nature (and hence the low conductivity) of the pristine film and additional tunneling barrier of the native oxide on the film interface [16].

After the FE-MISPC process, the second silicon layer is deposited on top of the initial film at 100°C in the thickness of about 200 nm (±30 nm). This deposition is done at the boundary conditions of amorphous and micro-crystalline silicon growth [17,18]. CS-AFM experiments are then again conducted on the previously processed areas for determining the impact of the second deposition on the FE-MISPC-induced features. Micro-Raman spectroscopy (diode laser, λ = 785 nm, *P *= 1 mW, objective 100×) is employed to characterize the crystallinity [19] of the FE-MISPC exposed spots after the second deposition.

In order to find the exposed areas after the second layer deposition, the samples were marked with a laser (HeCd laser, λ = 442 nm, *P *= 30 mW) prior to FE-MISPC process.

## Results

**Figure 1:**
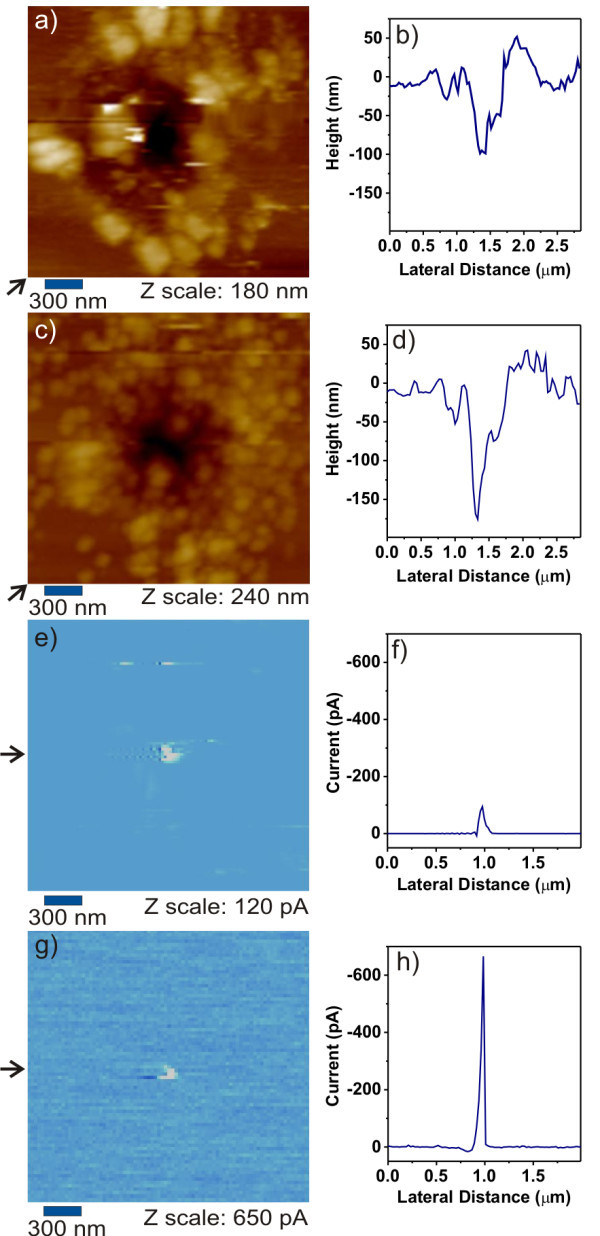
**Local topography images after**. **(a) **the FE-MISPC process and **(c) **the second deposition of the same spot. Their cross sections are plotted in **(b, d)**, respectively. **(e, g) **CS-AFM images corresponding to **(a) **and **(c)**, respectively. Their cross sections are plotted in **(f, h)**, respectively. Positions of the cross sections are indicated by arrows next to the images.

Figure 1a shows the typical local topography after an FE-MISPC experiment exhibiting current spikes over the set-point [12]. The diameter of the pit is 300 nm, and it can be seen that some material is accumulated around the hole. The cross section plotted in Figure 1b shows that the depth of the pit is 100 nm. The full-width-at-half-maximum (FWHM) is 200 nm. In Figure 1e is shown the local conductivity map of the same area obtained at the sample bias voltage of -25 V. The conductive region is mainly focused in the pit. The cross section plotted in Figure 1f shows the spatial profile of electrical current inside the pit. Peak current is 100 pA, and FWHM is 60 nm.

**Figure 2:**
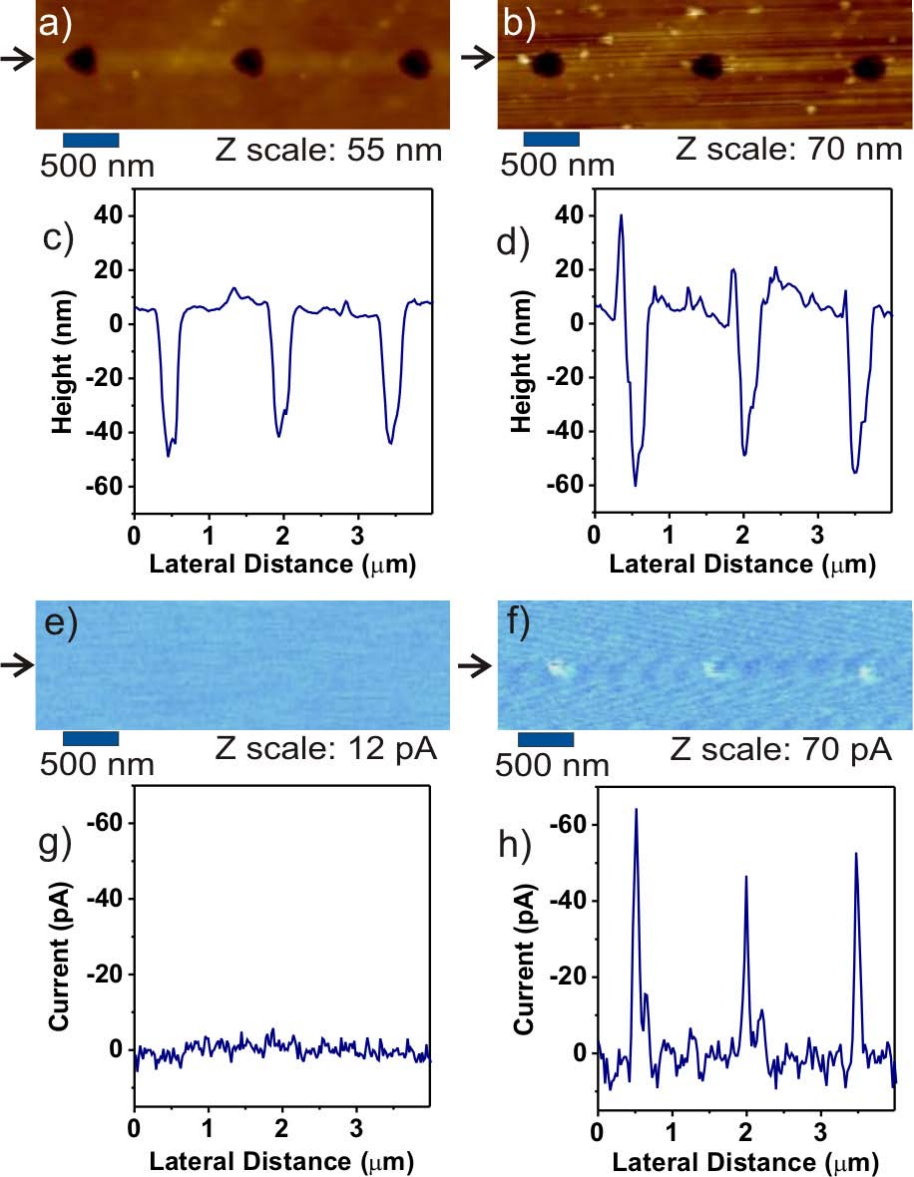
**Local morphology images after FE-MISPC resulting in non-conductive pits**. **(a) **AFM topography; **(e) **CS-AFM of the same spot, and their corresponding cross sections **(c, g)**; **(b) **AFM topography of the same area after the second deposition; **(f) **CS-AFM and the respective cross sections **(d, h)**. The cross sections are indicated by arrows next to the AFM images.

Figure 1c,g, shows the local topography and conductivity map obtained at the sample bias voltage of -25 V in exactly the same area as in Figure 1a,e after the second layer was deposited. AFM topography shows an accumulation of typical silicon micro- and nano-crystals [15] around the pit. CS-AFM shows conductive regions localized within the pit. Note that the individual silicon crystals present due to the second deposition do not appear conductive because the current pre-amplifier setting (sensitivity = 1 nA/V) was adjusted to the magnitude of the current in the pit. Scanning the same area with higher current sensitivity (1 pA/V) showed conductivity on every single crystal seen in the topography. Cross sections plotted in Figure 2d,h, respectively, show that the pit depth is now 175 nm (FWHM is 200 nm) and that the conductive region exhibits an electrical current peak of 670 pA (FWHM is 30 nm).

Figure 2a illustrates the local topography of an area after three separate FE-MISPC experiments exhibiting stable current. The pits this time are non-conductive as seen in the corresponding CS-AFM image and its cross section (see Figure 2e,g). Their diameter is about 300 nm for all the pits. Their depth is 40-50 nm as shown by the spatial profile in Figure 2c. FWHM is about 200 nm (middle pit).

**Figure 3:**
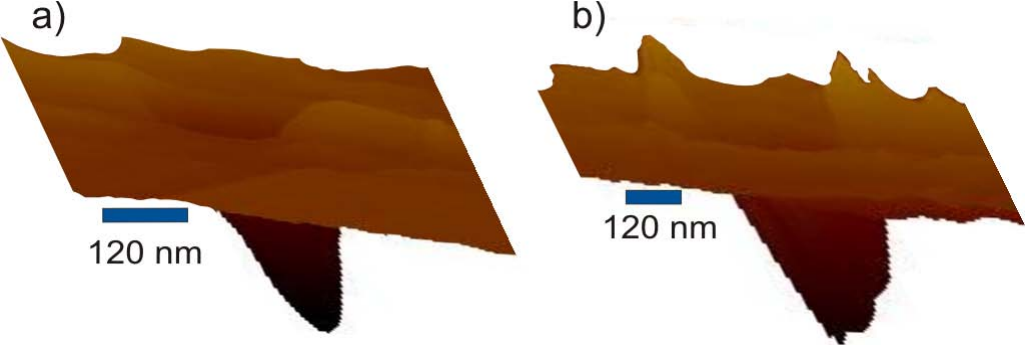
**Three-dimensional AFM topography of the middle pit in Figure 2**: **(a) **after FE-MISPC process, **(b) **after the second silicon deposition.

Topography of the same spot after second deposition (see Figure 2b) shows several small silicon nano-crystals scattered across the area. The depth of the pits increased to 50-60 nm as shown by the spatial profile in Figure 2d. FWHM is 180 nm (middle pit). In the CS-AFM image after the second deposition (see Figure 2f), it can be seen that the previously non-conductive pits now exhibit pronounced difference in conductance. Corresponding current spatial profile in Figure 3h shows a peak current up to 65 pA at -25 V. FWHM is 40 nm (middle pit).

Figure 3 shows the middle pit of Figure 2 in three-dimensional representation before (a) and after (b) the second deposition. Besides the growth-induced depth change, modifications in the local morphology inside the pit can also be seen. The bottom of the pit turns from smooth to rough. Note that the images of Figure 3a,b are optimized to emphasize on the features of the pit in the *z*-direction, and consequently their real aspect ratio is not maintained.

**Figure 4:**
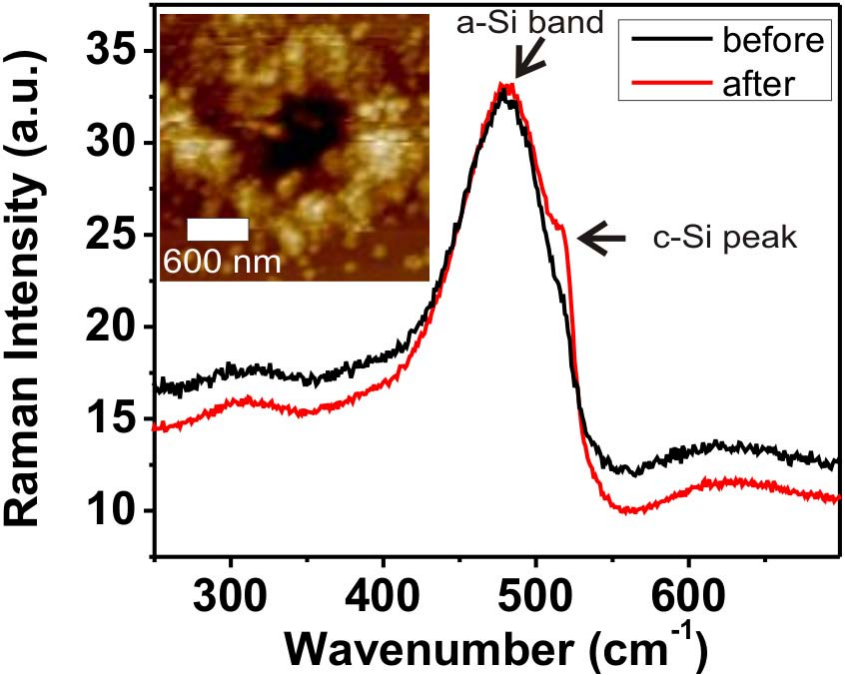
**Raman spectra of FE-MISPC induced conductive features before and after the second deposition process**. The inset shows the topography of the measured area corresponding to the spectrum "after". The spectrum was measured in the central part of the pit. Spectra are normalized to the amorphous band.

Figure 4 shows the micro-Raman spectrum measured on the conductive pit after second deposition (AFM topography is shown in the inset image). The crystalline silicon peak at 521 cm^-1 ^is well resolvable, even though it is superimposed with much more pronounced amorphous band. This is because most of the material in the focus of the Raman is amorphous. Accounting for Raman focus diameter of about 700 nm (objective 100×, λ = 785 nm) and crystalline region diameter of 100 nm, crystalline fraction makes only 2% of the detection area. Raman measurements, before the second deposition on various FE-MISPC-exposed spots, showed only broad amorphous band (typical spectrum shown in Figure 4), obviously because the crystalline phase amount was below the detection threshold.

## Discussion

The critical factor controlling the outcome of FE-MISPC is the AFM tip. When it is new, in the first few expositions, it produces larger, conductive pits irrespective of the exposition current. During those expositions, the tip is being "formed." After tip "forming," the use of exposition currents in the range of 0.05-0.15 nA results always in non-conductive pits as also reported previously [10]. Producing small conductive pits relies on current limitation [11] while allowing for current fluctuations [11,12]. The typical yield is 70% so far [12].

By correlating increased local conductivity [15] and crystalline silicon peak or at least a shoulder (because of <2% fraction of the detection area) in micro-Raman spectra, it can be concluded that silicon nanocrystals are formed inside the pits after the second deposition. This conclusion is also supported by the change of local morphology. As illustrated in Figure 3, the bottom of the pit changes from smooth to rough. Furthermore, the increase in the pit depth after the second deposition is smaller than the thickness of the deposited layer (changed by 75 nm in the case of conductive pits or by 10 nm the case of non-conductive pits vs. 200 nm of the second film thickness). This indicates that there must be some growth occurring inside the pits as well. This effect can be in particular pronounced because the second silicon deposition is performed at the boundary of amorphous and microcrystalline growth where silicon crystals typically protrude above the amorphous film because of their faster growth [15].

Under the boundary deposition conditions, silicon nanocrystals and their aggregates (the so-called microcrystalline columns) nucleate at random positions in otherwise uniform a-Si:H [15,17]. Upon using the localized FE-MISPC process, the nucleation became focused into the processed regions. In the case of initially conductive pits, the nanocrystal density is increased also around the pit compared to farther surroundings. This may be due to topographical as well as structural modification of the first a-Si:H film, because, e.g., some additional local stress and/or atomic scale defects may be induced around the processed area [20].

In the case of non-conductive pits, the overall density of nanocrystals remained uniform, i.e., nanocrystals are randomly scattered across the surface, except for the perfectly focused growth inside the pits. Formation of non-conductive pits introduces most likely less stress and defects in the local structure of the film, thus not enhancing crystal nucleation around the pit. The non-conductive pits exhibit pronounced increase in conductivity after the second deposition compared to the initial resistive state (see Figure 3). As the background exhibits conductivity of <5 pA (due to current pre-amplifier noise at the selected current range), the increase from the second deposition is of one order of magnitude or more. This indicates that new silicon nanocrystals are formed and localized in the pits. The non-conductive pits are thus the most promising candidates for selective growth of Si micro- and nano-crystals.

Note that the nanocrystals, which are scattered randomly across the surface or just around the pit, are also conductive compared to the a-Si:H background, in agreement with previous reports [15]. However, their conductivity is two orders of magnitude lower compared to the center of the pit. Hence, they do not appear as brighter dots in the current images. This is most likely because they are grown on the a-Si:H film (with possibly additional amorphous incubation layer [17]). It can be assumed that the much higher conductivity of the nanocrystals inside the pits is because they nucleate more readily without amorphous stage and are also better connected to the bottom electrode, e.g., via the conductive path made by the FE-MISPC process that may be further improved by the elevated temperature during the second deposition.

There are several possible factors that can promote nucleation and growth of silicon nanocrystals inside both types of the pits created by local FE-MISPC process. First, growth precursors during the second CVD deposition may become more localized inside the pits. Second, density of a-Si:H defects can be increased inside the pits due to local heating and/or evolution of hydrogen as in the case of laser annealing that also can promote further growth of crystalline silicon [20]. Third, local stress or strain may be increased inside the pits and may increase the nucleation probability. Fourth, crystal growth may proceed on the already existing crystals in the case of conductive pits. Fifth, the elevated temperature during second deposition (100°C) may also affect the crystallinity of the features. To resolve this, thermal annealing of a FE-MISPC-exposed sample was performed. The annealing conditions were identical to the second deposition conditions described above, but without the plasma. We noticed some increase in the local currents after the annealing only on the previously conductive pits. Since this temperature is not enough to promote Si deposition, this effect is merely thermal. In the case of non-conductive pits, there was no effect on the structural or electronic properties detected. The last two factors thus cannot explain the growth in non-conductive pits. The other factors may all contribute to certain extent, and the main contribution cannot be presently resolved.

## Conclusions

This study demonstrated that the deposition of a second silicon layer at the boundary condition of amorphous/micro-crystalline growth on top of the a-Si:H film could increase the conductivity of areas previously processed by the local FE-MISPC using AFM. The following effects were observed: (i) conductivity of conductive features (pits) was increased by up to six times, and (ii) new sub-100 nm conductive spots were generated in non-conductive pits. The increase in the local conductivity was attributed to the formation of silicon nanocrystals (<100 nm) inside the pits as evidenced by CS-AFM profiles. It was also corroborated by changes of morphology and by micro-Raman spectra. The process is the most defined in the case of non-conductive pits. This study thus opens perspectives for the growth of Si nanocrystals in predefined positions with nanoscale precision using the secondary deposition process. Such procedure, for instance, could be used to adjust the preferred properties of the nanocrystals by the deposition parameters.

## Abbreviations

AFM: atomic force microscopy; CS-AFM: current-sensing AFM; CVD: chemical vapor deposition; FE-MISPC: field-enhanced metal-induced solid phase crystallization; FWHM: full-width-at-half-maximum.

## Competing interests

The authors declare that they have no competing interests.

## Authors' contributions

EV carried out the AFM/CS-AFM measurements and drafted the manuscript. BR participated in the design and coordination of the study, and edited the manuscript. EŠ designed and materialized the exposition circuit and the control software. JS performed the CVD deposition of the silicon thin films. ML performed the Raman meaurements. JK concieved the study and participated in its coordination.

## References

[B1] Rezek B Nebel CE Stutzmann M "Polycrystalline Silicon Thin Films Produced by Interference Laser Crystallization of Amorphous Silicon" Jpn J Appl Phys 1999 38 L1083 10.1143/JJAP.38.L1083

[B2] Nakazawa K "Recrystallization of amorphous silicon films deposited by low-pressure chemical vapor deposition from Si_2_H_6 _gas" J Appl Phys 1991 69 1703 10.1063/1.347215

[B3] Lam LK Chen S Ast DG "Kinetics of nickel-induced lateral crystallization of amorphous silicon thin-film transistors by rapid thermal and furnace anneals" Appl Phys Lett 1999 74 1866 10.1063/1.123695

[B4] Fojtik P Dohnalová K Mates T Stuchlík J Gregora I Chval J Fejfar A Kočka J Pelant I "Rapid crystallization of amorphous silicon at room temperature" Philos Mag B 2002 82 1785 10.1080/13642810208222940

[B5] Yoon SY Park SJ Kim KH Jang J "Metal-induced crystallization of amorphous silicon" Thin Solid Films 2001 383 34 10.1016/S0040-6090(00)01790-9

[B6] Trojánek F Neudert K Bittner M Malý P "Picosecond photoluminescence and transient absorption in silicon nanocrystals" Phys Rev B 2005 72 075365

[B7] Fejfar A Mates T Čertík O Rezek B Stuchlík J Pelant I Kočka J "Model of electronic transport in microcrystalline silicon and its use for prediction of device performance" J Non-Cryst Solids 2004 338 303 10.1016/j.jnoncrysol.2004.02.063

[B8] Tan YT Kamiya T Durrani ZAK Ahmed H "Room temperature nanocrystalline silicon single-electron transistors" J Appl Phys 2003 94 633 10.1063/1.1569994

[B9] Bisi O Ossicini S Pavesi L "Porous silicon: a quantum sponge structure for silicon based optoelectronics" Surf Sci Rep 2000 38 1 10.1016/S0167-5729(99)00012-6

[B10] Rezek B Šípek E Ledinský M Krejza P Stuchlík J Kočka J "Spatially localized current-induced crystallization of amorphous silicon films" J Non-Cryst Solids 2008 354 2305 10.1016/j.jnoncrysol.2007.10.045

[B11] Rezek B Šípek E Ledinský M Stuchlík J Vetushka A Kočka J "Creating nanocrystals in amorphous silicon using a conductive tip" Nanotechnology 2009 20 045302 10.1088/0957-4484/20/4/045302 19417314

[B12] Verveniotis E Rezek B Šípek E Stuchlik J Kočka J "Role of current profiles and AFM probes in electric crystallization of amorphous silicon" Thin Solid Films 2010 518 5965 10.1016/j.tsf.2010.05.107

[B13] Luterová K Pelant I Fojtík P Nikl M Gregora I Kočka J Dian J Valenta J Malý P Kudrna J Štěpánek J Poruba A Horváth P "Visible photoluminescence and electroluminescence in wide-band gap hydrogenated amorphous silicon" Philos Mag B 2000 80 1811

[B14] Rezek B Mates T Stuchlík J Kočka J Stemmer A "Charge storage in undoped hydrogenated amorphous silicon by ambient atomic force microscopy" Appl Phys Lett 2003 83 1764 10.1063/1.1606872

[B15] Rezek B Stuchlík J Fejfar A Kočka J "Microcrystalline silicon thin films studied by atomic force microscopy with electrical current detection" J Appl Phys 2002 92 587 10.1063/1.1486032

[B16] Vetushka A Feifar A Ledinský M Rezek B Stuchlik J Kočka J "Comment on "Current routes in hydrogenated microcrystalline silicon"" Phys Rev B 2010 81 237301 10.1103/PhysRevB.81.237301

[B17] Kim SK Lee HH "Intrinsic phase boundary between amorphous and crystalline structures for chemical vapor deposition" J Cryst Growth 1995 151 200 10.1016/0022-0248(95)00017-8

[B18] Kočka J Fejfar A Mates T Fojtík P Dohnalová K Luterová K Stuchlík J Stuchlíková H Pelant I Rezek B Stemmer A Ito M "The physics and technological aspects of the transition from amorphous to microcrystalline and polycrystalline silicon" Phys Status Solidi C 2004 1 1097

[B19] Ledinský M Vetushka A Stuchlík J Mates T Fejfar A Kočka J Štěpánek J "Crystallinity of the mixed phase silicon thin films by Raman spectroscopy" J Non-Cryst Solids 2008 354 2253

[B20] Ivlev G Gatskevich E Cháb V Stuchlík J Vorlíček V Kočka J "Dynamics of the excimer laser annealing of hydrogenated amorphous silicon thin films" Appl Phys Lett 1999 75 498 10.1063/1.124428

